# Identification of linear epitopes in SjSP-13 of *Schistosoma japonicum* using a GST-peptide fusion protein microplate array

**DOI:** 10.1186/s13071-019-3767-2

**Published:** 2019-10-30

**Authors:** Li Ma, Wenrong Zhao, Xunya Hou, Mengmeng Liu, Yanna Li, Li Shen, Xindong Xu

**Affiliations:** 1 Research Center for Translational Medicine, Shanghai East Hospital, Tongji University School of Medicine, Shanghai, China; 2 Hunan Institute of Parasitic Diseases, Yueyang, China

**Keywords:** Epitope, *Schisitosoma japonicum*, Diagnosis, SjSP-13, Fusion protein

## Abstract

**Background:**

The identification and characterization of epitopes facilitate the discovery and development of new therapeutics, vaccines and diagnostics for infectious diseases. In this study, we developed a glutathione S-transferase (GST)-peptide fusion protein microplate array for the identification of linear B-cell epitopes and applied this novel method to the identification of linear B-cell epitopes of SjSP-13, an immunodiagnostic biomarker of schistosomiasis japonica.

**Methods:**

SjSP-13 was divided into 17 overlapped peptides (p1-17), and the coding sequence of each peptide was obtained by annealing two complementary oligonucleotides. SjSP-13 peptides were expressed by fusion with an N-terminal GST tag and a C-terminal 6xHis tag. The GST-peptide-His fusion protein was specifically bound to the Immobilizer Glutathione MicroWell 96-well plates without purification. SjSP-13 peptides and core epitopes that could be recognized by sera from schistosomiasis patients were identified by ELISA and confirmed by Western blot analysis. The receiver operating characteristic (ROC) analysis was performed to determine the diagnostic validity of the identified peptide.

**Results:**

Full-length GST-peptide-His fusion proteins were successfully expressed and specifically bound to the Immobilizer Glutathione MicroWell 96-well plates. Two adjacent peptides (p7 and p8) were found to be highly immunogenic in humans. The core epitope of p7 and p8 is an 11-aa peptide (_80_KCLDVTDNLPE_90_) and an 8-aa peptide (_90_EKIIQFAE_97_), respectively. The area under the ROC curve (AUC) value of the peptide which contains the two identified epitopes is 0.947 ± 0.019. The diagnostic sensitivity and specificity of the peptide is 76.7% (95% CI: 68.8–84.5%) and 100%, respectively.

**Conclusions:**

_90_EKIIQFAE_97_ and _80_KCLDVTDNLPE_90_ are the two linear epitopes of SjSP-13 recognized by patient sera, and could be potential serological markers for schistosomiasis japonica.
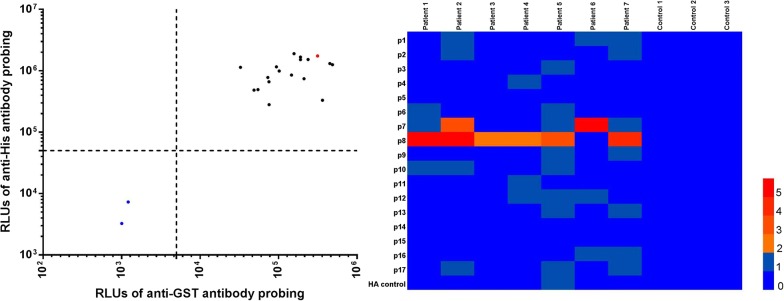

## Background

Schistosomiasis is an important neglected tropical disease (NTD), which remains a serious public health problem in the tropics and subtropics with more than 230 million people infected in 78 countries [1, 2]. There are three major schistosome species that infect humans: *Schistosoma japonicum*, *S. mansoni* and *S. haematobium*. Of the three species, *S. japonicum* which is mainly found in China, the Philippines and small pockets of Indonesia, is recognized as the most difficult to control because of its zoonotic nature [3, 4]. The implementation of the new integrated strategy with emphasis on control of the infection source across China since 2004 has greatly reduced *S. japonicum* in humans, livestock, and intermediate host *Oncomelania hupensis* snails. It has been estimated that there were more than 38,000 cases of schistosome infections in 2017. Moreover, the control of schistosomiasis in China is particularly challenging due to the wide distribution of its snail hosts and the wide range of domestic and wild mammals that act as reservoirs for human infection. Thus, schistosomiasis remains one of the most important public health problems in China.

The scarcity of an effective diagnostic method is one of the factors that contribute to the prevalence of schistosomiasis [5]. Additionally, the current schistosomiasis elimination plan in China highlights the importance of the development of sensitive diagnostic techniques as the treatment of targeted populations is a major strategy [6]. However, the sensitivity of traditional parasitological methods, such as stool examination, is poor in low endemic areas [7, 8]. Immunodiagnostic techniques are promising tools for detecting mild-to-moderate infections. However, the currently available immunodiagnostic assays have low specificity because of the use of crude antigens, such as soluble egg antigens (SEA) consisting thousands of parasite antigens, presenting a wide cross-reaction with antigens from other worms [9, 10]. Thus, it is a prerequisite to select diagnostic biomarkers with high sensitivity and specificity.

Recently, several novel proteins with high immunogenicity were identified *via* immunomics [11–13], including SjSP-13, an immunodiagnostic marker of schistosomiasis japonica [14]. SjSP-13 is a member of a multigene family of saposin-like proteins, which contains the SAP-B domain that is characterized by six cysteine residues forming disulfide bonds to stabilize its structure [15]. In *S. japonicum*, this multigene family is present in the form of 15 members (SjSAPLP1 (SjSP-13) to SjSAPLP15) [16]. These proteins exhibit a potent lytic activity on human erythrocytes and peripheral blood mononuclear cells. Like SjSP-13, SjSAPLP4, and SjSAPLP5 are candidate diagnostic markers for schistosomiasis japonica [16]. SjSP-13 is highly expressed in young worms and adult worms but poorly expressed in eggs [17]. In adult worms, SjSP-13 is abundantly distributed on the surface and lumen of the esophageal and intestinal tracts [16]. In addition, SmSP-13, the homologous gene of SjSP-13 in *S. mansoni*, has been found in worm vomitus [18]. However, the epitope of this immunodominant antigen is still unclear.

In this study, we developed a novel GST-peptide fusion protein microplate array for mapping the linear B-cell epitopes. The epitopes of SjSP-13 were identified by this technology.

## Methods

### Peptide mapping

SjSP-13 protein contains 177 amino acids (aa), of which 21 aa at the N-terminal are predicted to be a signal peptide. We divided a 156-aa length SjSP-13 fragment without the signal peptide into 17 peptides with a length of 18 aa per peptide, except for the last 12-aa long peptide. There were 9 overlapped amino acids between the two adjacent peptides. The coding DNA sequences of each peptide were obtained by annealing two single-stranded oligonucleotides with complementary sequences. *BamH*I and *Xho*I sticky terminals were added in the upstream and downstream oligonucleotides, respectively. The resulting DNA fragments were inserted into a pGEX-His vector. The GST-peptide-His tag fusion protein was expressed in the *E. coli* BL21 strain with 1mM Isopropyl-D-1-thiogalactopyranoside (IPTG) induction. Cells were lysed by B-Per (Pierce, Rockford, USA) and treated with the recommended concentration of DNase, RNase and PMSF. The whole *E. coli* lysate without centrifugation was directly dissolved in 8M urea overnight at room temperature. After centrifugation, proteins in supernatant were renatured in refolding buffer (1.0 mM TCEP, 250 mM NaCl, 12.5 mM β-cyclodextrin, 50 mM Tris-HCl, pH 8.5). The refolded proteins were stored at − 20 °C until use.

### Western blot

Proteins were separated by SDS-PAGE and then transferred onto a nitrocellulose membrane. Western blot was performed using anti-GST tags (Abmart, Shanghai, China), anti-6xHis tags (Abmart) and schistosomiasis patient serum as the primary antibodies. Anti-mouse (Promega, Madison, USA) and anti-human (Promega) IgG horseradish peroxidase (HRP)-linked whole antibodies were used as the secondary antibodies. The ECL-PLUS system (Pierce) was used for detection according to the manufacturer’s instructions.

### Serum collection and adsorption

Ninety-seven infected human serum samples were collected from villagers living in schistosomiasis-endemic areas who were diagnosed as schistosomiasis patients using the Kato-Katz method [19]. The egg counts of these patients ranged from 8 to 320 fecal eggs per gram (epg). The sera of healthy humans were used as controls.

Seven infected sera with high antibody titres to SjSP-13 and three control sera were selected for epitope screening. Sera were incubated with *E. coli* extracts and the GST protein to remove the corresponding antibodies before use. A mixture of 1 ml human serum, 5 ml bacterial lysate, 0.1 ml GST bound glutathione sepharose 4B beads and 3.9 ml PBS (pH 7.4) was rocked for 5 h at room temperature. After centrifugation, absorbed serum was stored at − 20 °C until use.

### Preparation of GST-peptide-His fusion protein microplate array

The refolded GST-peptide-His fusion proteins were diluted in PBS with a final concentration of 50–100 μg/ml. Immobilizer Glutathione MicroWell plates (Nunc, Denmark) were coated with 100 µl/well of fusion protein solution overnight at 4 °C. Recombinant GST-His solution, *E. coli* lysate and PBS were set as positive, negative and blank controls, respectively. To remove the unbound proteins, the plates were washed five times with 150 mM PBS containing 0.05% Tween-20 (PBST) and blocked with PBST plus 5% skim milk powder overnight at 4 °C. After washing five times, the 96-well microplate bound GST-peptide-His fusion protein was sealed and stored at − 20 °C before use.

To ensure the integrity of the bound GST-peptide-His fusion protein on the 96-well microplates, 100 μl of 1:2,000 diluted anti-GST and anti-6xHis tag were added to each well of the microplate for 1 h incubation at 37 °C. After washing for five times, 100 μl of 1:20,000 diluted HRP conjugated anti-mouse IgG secondary antibody (Promega) was added to wells for a further 1-h incubation at 37 °C. Wells were washed five times before 100 μl of SuperSignal ELISA Femto Maximum Sensitivity Substrate (Pierce) was added. The bound antibodies were quantified by measuring the relative light units (RLUs) at 425 nm with a luminometer between 1–5 min after adding the substrate (Molecular Devices, San Jose, USA).

### B-cell linear epitope identification

The B-cell linear epitopes of SjSP-13 were identified by ELISA using the 96-well microplate bound GST-peptide-His fusion protein. The 1:100 diluted adsorbed human sera were added to the microplate and 1:20,000 diluted HRP conjugated anti-human IgG secondary antibody (Promega) was used as the detecting antibody. The ratio of RLUs of a human serum sample to GST-peptide-His fusion protein was calculated using the formula: R = (RLUs of GST-peptide-His fusion protein − RLUs of PBS)/ (RLUs of GST-His − RLUs of PBS), and a positive reaction was considered when R ≥ 2. The screened epitopes were further verified by Western blot.

### Structure prediction

Swiss-model structure online analysis software was used to simulate the three-dimensional structure model of SjSP-13 protein and the highest score was selected as the alignment template [20]. PyMOL software was then used for analyzing the positional structure of the epitope on the SjSP-13 protein [21].

### ELISA

SjSP-13-based ELISA was performed as described previously [22]. Peptide-based ELISA was performed on synthesized peptides (Sangon Biotech, Shanghai, China) consisting of the two adjacent epitopes identified in this study. Polystyrene 96-well microtiter plates (Nunc, Roskilde, Denmark) were coated with peptide (5 μg per ml in coating buffer; 100 μl per well) overnight at 4 °C. Wells were blocked with PBST containing 5% non-fat milk (PBST-milk) for 2 h at 37 °C. Serum samples were diluted in 1:50 with blocking buffer and incubated for 1 h at 37 °C. HRP-conjugated goat anti-human IgG (Promega) was used to detect antibodies bound to peptide. Enzymatic reactions were developed by adding 100 μl TMB substrate to each well and terminated by stop reagent (50 μl 2 N H_2_SO_4_ per well). Absorbance values were measured at 450 nm. ELISA readings were done in duplicates.

### Data analysis

Heat maps were performed with R statistical software. OD_450 nm_ value was expressed as the mean ± standard error. The differences of OD_450 nm_ between groups were analyzed by Student’s t-test. The diagnostic accuracy was evaluated by receiver operating characteristic (ROC) curve analysis. The area under the ROC curve (AUC) was calculated to assess the overall diagnostic performance. The sensitivity and specificity of various diagnostic tools were compared by chi-square test or Fisher’s exact test. A *P-*value < 0.05 was considered statistically significant.

## Results

### SjSP-13 peptide library construction

**Fig. 1:**
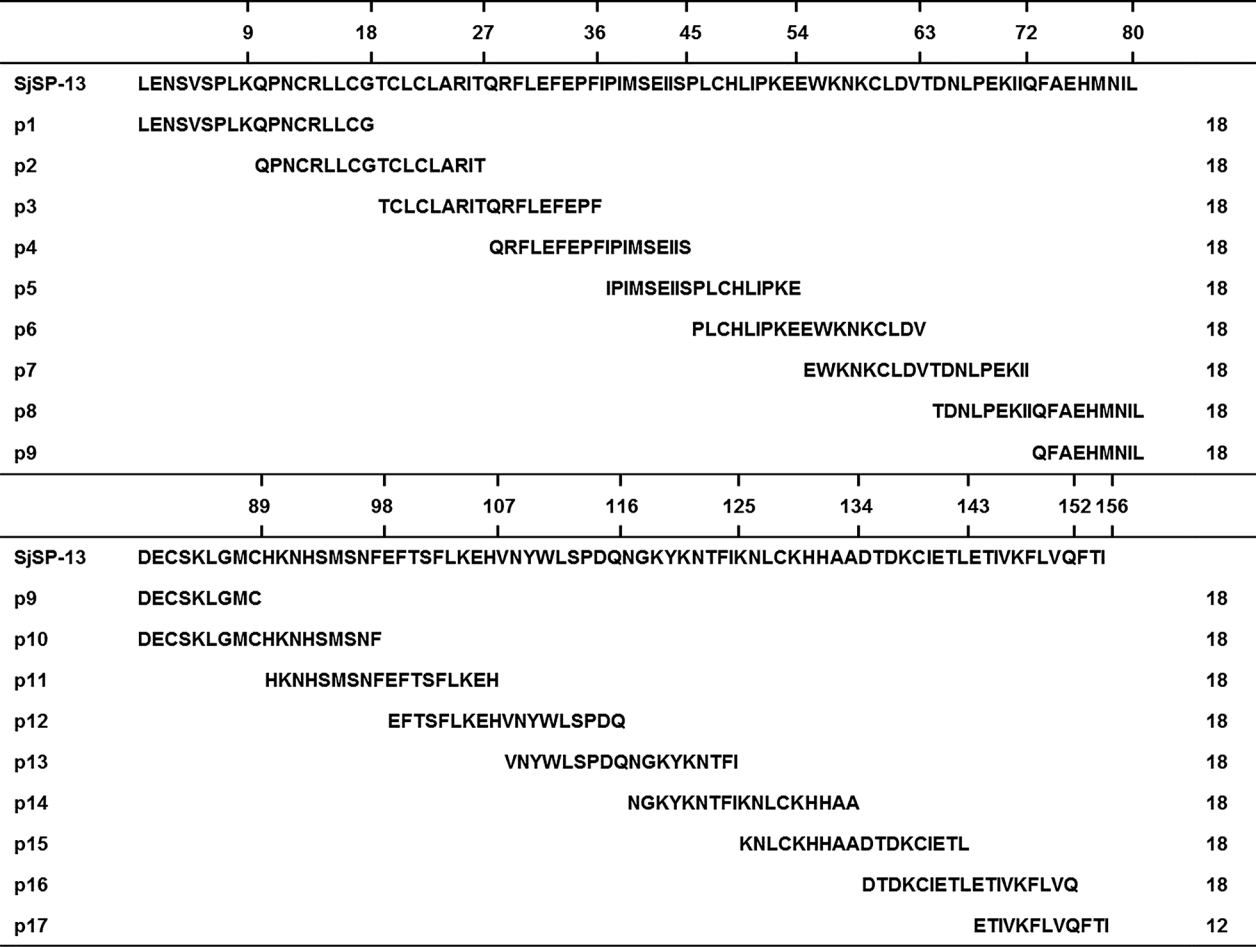
Peptide mapping of SjSP-13. 156-aa length SjSP-13 fragment without the signal peptide was divided into 17 peptides with the length of 18 aa per peptide, except for the last 12-aa length peptide. There are 9 overlapped amino acids between the two adjacent peptides

A 156-aa length SjSP-13 without signal peptide was divided into 16 18-aa length peptides and a 12-aa length peptide, namely p1 to p17. There are 9 overlapped amino acids between the two adjacent peptides (Fig. 1). The coding DNA sequences of each peptide were cloned into a pGEX-His vector, a modified GST fusion protein expression vector with an additional C-terminal 6xHis tag (see Additional file 1: Figure S1a). The full-length expression of GST-peptide-His fusion protein was confirmed by Western blot with anti-GST and anti-His mAb (see Additional file 1: Figure S1b). Since most of the fusion proteins are expressed as inclusion bodies, we directly dissolved the *E. coli* lysate in 8 M urea and then obtained soluble fusion proteins by dilution in protein refolding buffer.

### Construction of GST-peptide-His fusion protein microplate array

**Fig. 2:**
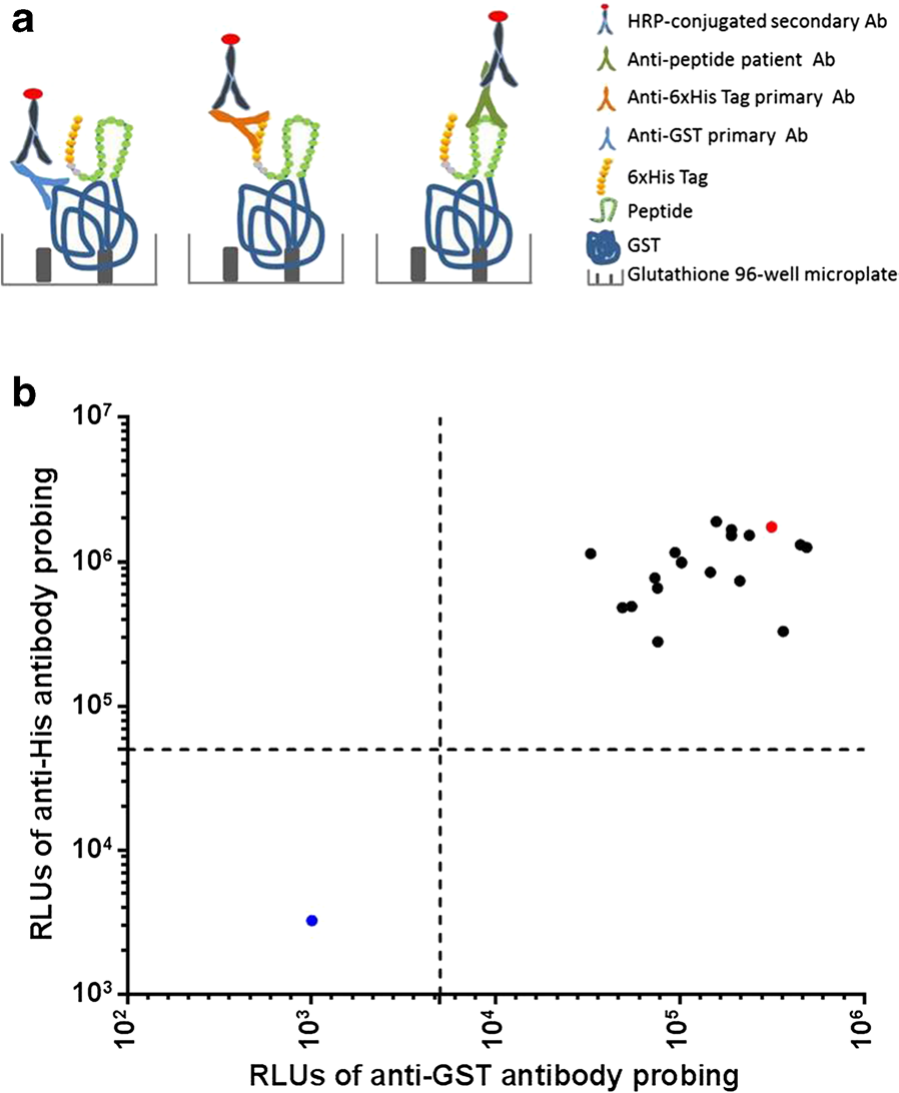
Schematic diagram of the GST-peptide-His fusion protein microplate array. **a** GST-peptide-His fusion protein was bound to the GSH-immobilized microplates *via* the interaction of GST and GSH. The integrity of the bound GST-peptide-His fusion protein could be detected by anti-GST and anti-6xHis antibodies. **b** High RLUs were detected by chemiluminescent ELISA when using both anti-GST and anti-6xHis antibodies, validating the reliability of the epitope screening platform. *Abbreviation*: RLUs, relative light units. *Key*: red dot, the positive recombinant GST protein with an N-terminal 6 x His tag (GST-His); blue dot, negative *E. coli* lysis; black dots, GST-peptide-His fusion proteins

We developed a GST fusion protein-based rapid B-cell linear epitope screening method (Fig. 2a). GST-peptide-His fusion protein was bound to the GSH-immobilized microplates *via* the interaction of GST and GSH. The binding fusion proteins were detected by anti-GST mAb and the integrity of fusion proteins was detected by anti-His tag mAb. As shown in Fig. 2b, the relative light units (RLUs) of all the refolded GST-peptide-His fusion proteins are significantly higher than those of the *E. coli* lysate control, indicating that the fusion proteins successfully refolded and the proteins bound on the plates were full-length.

### Screening SjSP-13 peptide recognized by schistosomiasis patient sera

**Fig. 3:**
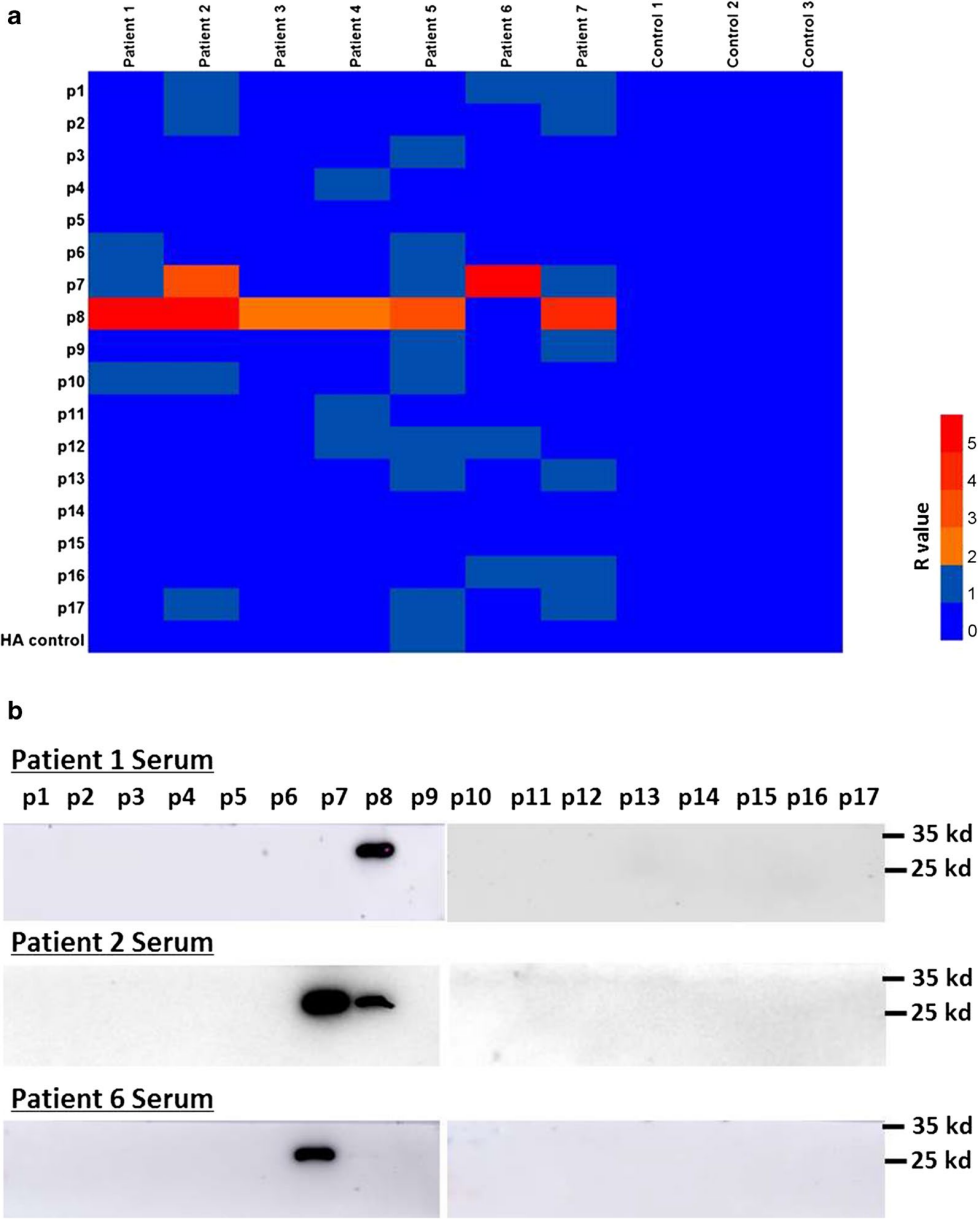
Identification of SjSP-13 peptides recognized in sera from schistosomiasis patients. **a** Reaction of 10 human serum samples (seven infected samples and three uninfected samples from healthy individuals) to the 17 GST-peptide-His fusion proteins determined by chemiluminescent ELISA. R = (RLUs of GST-peptide-His – RLUs of PBS)/ (RLUs of GST-His – RLUs of PBS). GST-peptide-His with R ≥ 2 were considered as seropositive reaction. *Abbreviations*: RLUs, relative light units; HA, hemagglutinin tag-GST fusion protein. **b** The recognition of p7 and p8 by the representative serum samples of Patients 1, 2 and 7 was confirmed by Western blot

The interaction between SjSP-13 peptides and schistosomiasis patient serum samples was evaluated by chemiluminescent ELISA. For initial screening of the 17 SjSP-13 peptides, we used ten serum samples from seven infected individuals and three uninfected individuals as controls. The result indicates that p8 could be recognized by 6 serum samples from Patients 1, 2, 3, 4, 5 and 7, and p7 could be recognized by 2 serum samples from Patients 2 and 6. None of the 17 SjSP-13 peptides react to the uninfected serum (Fig. 3a). We then selected representative serum samples of Patient 1, 2 and 7 for verification by Western blot. The results are consistent with ELISA (Fig. 3b).

### Identification of SjSP-13 B-cell linear core epitope

**Fig. 4:**
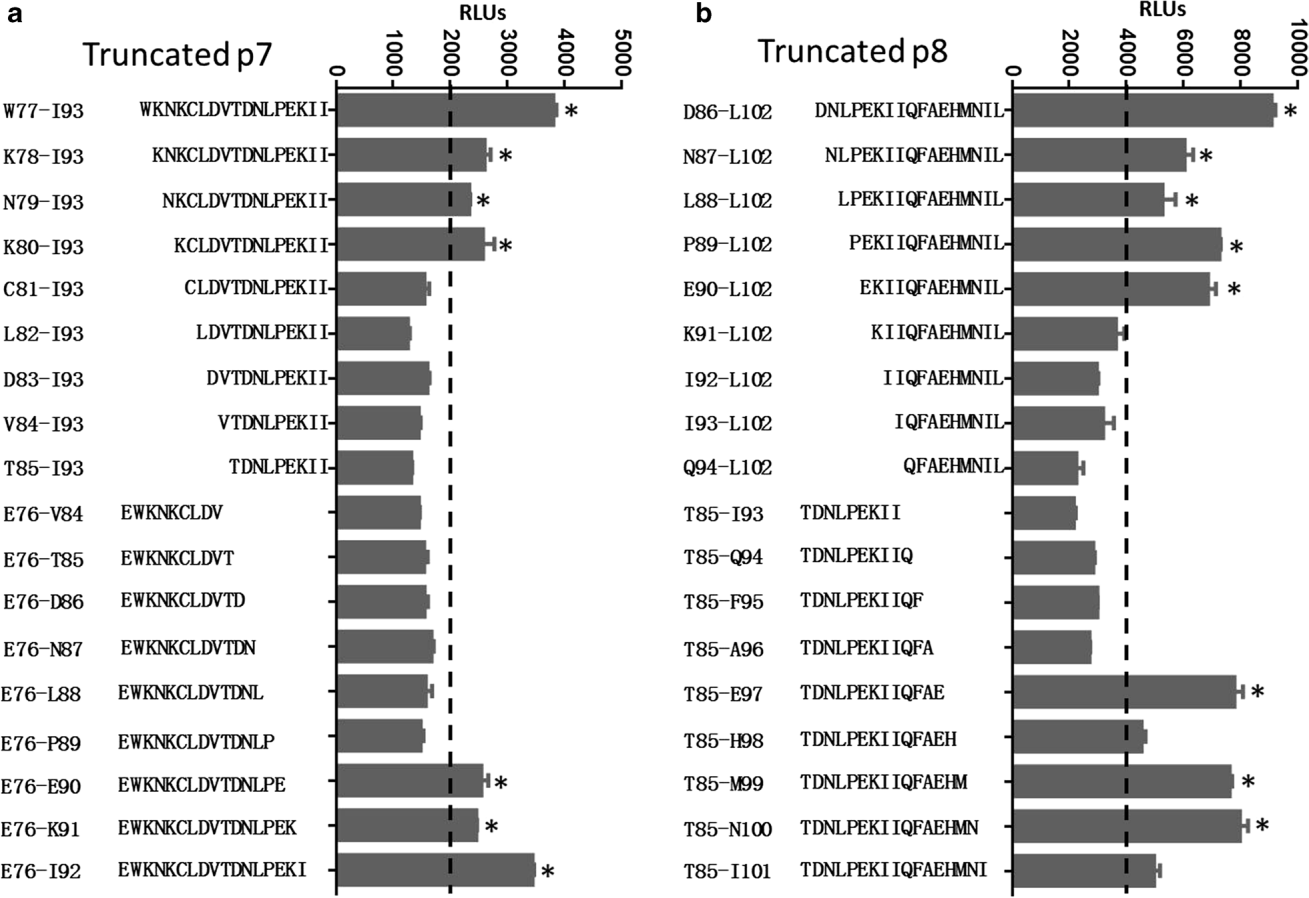
Core epitope identification. The core epitope in p7 and p8 was identified using a panel of truncated fusion peptides expressed with GST and 6xHis tag. **a** The core epitope of p7 is an 11-aa peptide _80_KCLDVTDNLPE_90_, spanning positions K80 to E90. **b** The core epitope of p8 is an 8-aa peptide _90_EKIIQFAE_97_, spanning positions E90 to E97. *P < 0.05 when compared with negative *E. coli* lysis using a Student’s t-test

We defined the core epitope in p7 and p8 using a panel of truncated peptides spanning the region of p7 and p8 by chemiluminescent ELISA as above (Fig. 4). For p8, progressive truncation of the peptide resulted in loss of binding effect to antibody when the peptide lost E90 at the N-terminal and E97 at the C-terminal. Therefore, the core epitope of p8 is an 8-aa peptide EKIIQFAE, spanning positions E90 to E97 (Fig. 4a). For p7, truncated peptides lose the binding effect when K80 at the N-terminal and E90 at the C-terminal are removed. The sequence of p8 core epitope spans K80 to E90, an 11-aa peptide KCLDVTDNLPE (Fig. 4b).

### Three-dimensional visualization of immunogenic epitopes

**Fig. 5:**
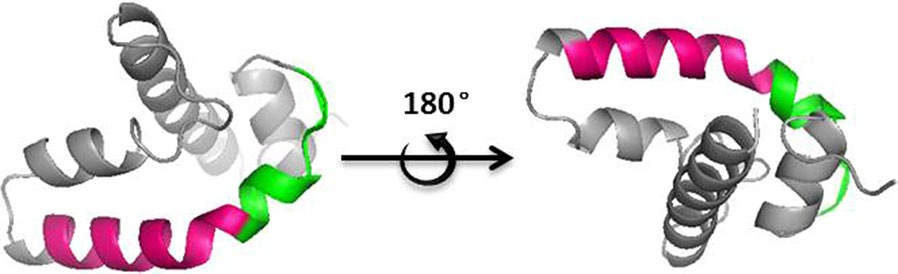
Visualization of immunogenic epitopes in the tertiary structure of saposin-like domain of SjSP-13. The alignment modeling sequence of SjSP-13 was from K50 to K133. _90_EKIIQFAE_97_ is a loop surface epitope, while _80_KCLDVTDNLPE_90_ is an α-helix surface epitope. *Key*: red, _80_KCLDVTDNLPE_90_; green, _90_EKIIQFAE_97_

The three-dimensional structure of the SjSP-13 protein was predicted by a Swiss-model server. The alignment template SMTL ID was 5fi9.2.A, Sphingomyelin phosphodiesterase, which is a 2-subunit lipase; the smaller subunit is a member of the saposin-like (SAPLIP) proteins and the larger subunit is a GDSL lipase. The alignment modeling sequence of SjSP-13 was from K50 to K133. It is predicted that five amino acid residues (IQFAE) of the epitope _90_EKIIQFAE_97_ are located on the exposed random crimp segment and the other three in an α-helix, while the whole sequence of epitope _80_KCLDVTDNLPE_90_ is located in an α-helix (Fig. 5).

### Diagnostic validity of SjSP-13 peptide

**Fig. 6:**
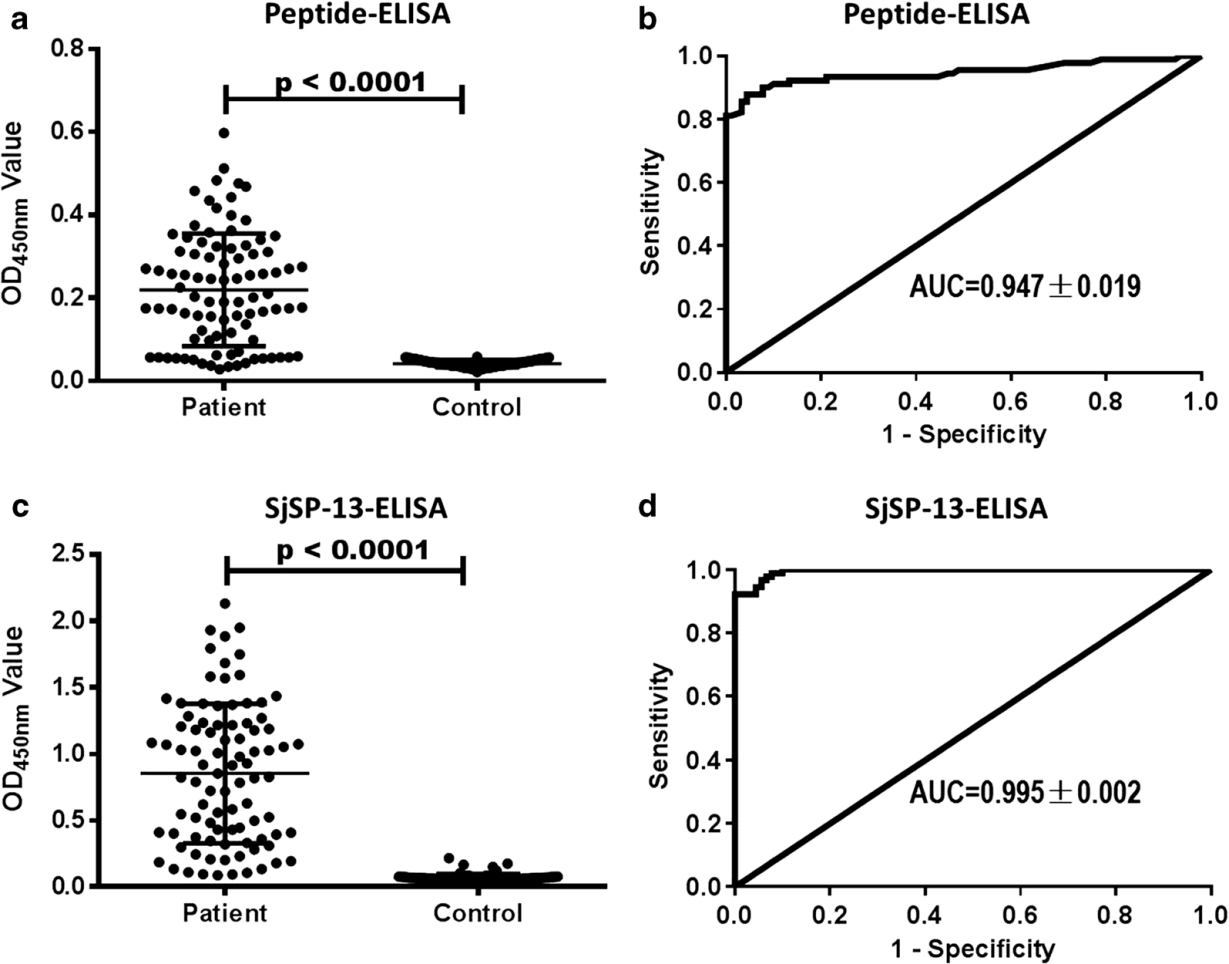
Comparison of the diagnostic performance of peptide-ELISA and SjSP-13-ELISA. The specific antibody to peptide (**a**) and SjSP-13 (**c**) in patients (*n* = 90) and healthy controls (*n* = 90) were evaluated by ELISA. ROC analysis was performed for peptide (**b**) and SjSP-13 (**d**). Data were compared using a Student’s t-test

A 27-aa SjSP-13 peptide (EWKNKCLDVTDNLPEKIIQFAEHMNIL) which contains the two adjacent epitopes was applied for detecting schistosome infection. The specific antibody levels of the peptide in patients were significantly higher than healthy controls (*t*_(89)_ = 13.24, *P* < 0.0001) (Fig. 6a). The ROC analysis was performed to determine the diagnostic validity of the peptide. As shown in Fig. 6b, the AUC value of the peptide is 0.947 ± 0.019. We defined the cut-off value as 2.1 times the mean OD_450 nm_ value of the healthy control serum. The diagnostic sensitivity and specificity of the peptide is 76.7% (95% CI: 68.8–84.5%) and 100%, respectively (Table 1). We also performed SjSP-13 ELISA using sera from the same cohort (Fig. 6c). The AUC value of SjSP-13 is 0.995 ± 0.002 (Fig. 6d). The sensitivity of SjSP-13 [92.2% (95% CI: 86.3–98.1%)] is significantly higher than SjSP-13 peptide, but the specificity of SjSP-13 [95.6% (95% CI: 91.6–99.5%)] is slightly but not significantly lower than SjSP-13 peptide (Table 1). In addition, we examined the cross-reaction of the peptide to patients with clonorchiasis (*n* = 38). The OD_450 nm_ value range was 0.022–0.058, much lower than the cut-off value (0.105). No cross-reaction was observed.

**Table 1:** Comparison of diagnostic validity of SjSP-13 peptide with SjSP-13 recombinant protein

Antigen	Patients(*n* = 90)			Healthy controls (*n* = 90)		
	True positive	False negative	Sensitivity (%) (95% CI)*	True negative	False positive	Specificity (%) (95% CI)
Peptide	69	21	76.7 (68.8–84.5)	90	0	100 (100–100)
SjSP-13	83	7	92.2 (86.3–98.1)	86	4	95.6 (91.6–99.5)

* *χ*^2^ = 8.289, *df* = 1, *P* = 0.004 when compared by a chi-square test

## Discussion

The global schistosomiasis control programme has made tremendous progress in reducing the prevalence of disease and morbidity in many endemic areas mainly due to the widespread treatment with the anti-schistosome agent praziquantel. However, high reinfection rates coupled with the risk of drug resistance support the need for new interventions, such as vaccination. Previous studies have identified a highly immunogenic antigen, SjSP-13, a member of a multigene family of saposin-like proteins. This antigen could be widely recognized by serum IgG of infected humans and has been applied for immunodiagnosis of schistosomiasis [14, 22]. Due to their highly immunogenic properties, B-cell epitopes of SjSP-13 were identified in this study.

B-cell epitopes can be divided in two categories: continuous and discontinuous. Continuous or linear epitopes are made up of consecutive amino acids, whereas discontinuous or conformational epitopes constitute spatially folded amino acids. The prediction of B-cell epitopes is much more complex than the prediction of T-cell epitopes and conformational epitopes. This might be due to the reason that linear B-cell epitope possesses variable lengths, from 2 to 85 amino acids, compared to the almost fixed length core of T-cell epitopes [23]. This variability imposes several obstacles for algorithm developers. The core sequence linear epitope is commonly shorter than the predicted peptide and should be verified experimentally by truncated or residue substitution peptides. Such experimental methods for identification of B-cell epitopes are costly and time-consuming.

Here, we developed a GST-peptide fusion protein microplate array, in which each peptide was expressed with a C-terminal GST tag and an N-terminal 6xHis tag, and then arrayed in GSH-immobilized plates through the interaction of GST with its substrate glutathione. The binding amount and integrity of fusion proteins could be assessed by anti-GST and anti-6xHis tag Abs. This technique was much more economical compared to identifying epitopes by synthetic peptides, as the cost of synthesized oligonucleotides is cheaper. Unlike synthetic peptides, the peptides in the form of a GST fusion protein could be easily verified by Western blot analysis. In addition, the GST-peptide fusion proteins can be used in other experiments, such as GST pull-down. In this study, we divided SjSP-13 into 17 peptides with nine overlapped amino acids between the two adjacent peptides. Two peptides, p7 and p8, could be recognized by schistosomiasis patient sera specifically.

We further determined the core epitopes of these two peptides. The core epitope of p7 is an 11-aa peptide, _80_KCLDVTDNLPE_90_, and that of p8 is an 8-aa peptide, _90_EKIIQFAE_97_. Interestingly, these two epitopes consist of predominantly hydrophobic residues in the center flanked by charged amino acids, consistent with general B-cell epitopes [24]. The two core epitopes are adjacent to the SjSP-13 primary structure, with an overlapped residue E_90_. _90_EKIIQFAE_97_ is a loop surface epitope, while _80_KCLDVTDNLPE_90_ is an α-helix surface epitope. We observed _90_EKIIQFAE_97_ could be recognized by six out of seven patients’ sera, while only two patients’ sera interacted with _80_KCLDVTDNLPE_90_. The creation of bends and flexibility of _90_EKIIQFAE_97_ might be responsible for more efficient presentation and recognition than _80_KCLDVTDNLPE_90_ in human immune cells.

We also compared the diagnostic performance of a peptide containing the two adjacent epitopes with SjSP-13 recombinant protein. Although peptide-ELISA has a 100% of specificity, the sensitivity is reduced 15.5% when compared with SjSP-13. The finding of the SjSP-13-positive but peptide-negative patients indicates that some epitopes were missed in our screening study. The variation of epitope recognition in different individuals may be linked to genetic polymorphisms of the human HLA class II alleles. In fact, there is evidence that the HLA alleles play crucial roles in modulation of the immune response to schistosome infection [25–27]. In addition, the polymorphism of SjSP-13 gene may be the other factor explaining the reduction of sensitivity of peptide-ELISA. The peptide sequence of the two epitopes identified in the present study was highly polymorphic according to our previous study [22].

The early diagnostic performance of SjSP-13 recombinant protein was evaluated in animal models previously [17]. SjSP-13 specific antibodies could be detected as early as 3 weeks post-infection in mouse model. However, the increase of SjSP-13 specific antibodies was not observed in rabbit model at this time point. These results indicate that, like SjSP-13 protein, SjSP-13-derived epitopes were not ideal biomarkers for detection of early *S. japonicum* infection.

## Conclusions

This study identified two linear B-cell epitopes of SjSP-13, an immunodominant antigen of *S. japonicum via* a novel linear B-cell epitope screening method based on a GST-peptide fusion protein microplate array. The high immunogenicity of the two epitopes of SjSP-13 in humans makes them promising candidates for diagnosis of schistosomiasis japonica.

## Supplementary information

**Additional file 1: Figure S1.** Construction of GST-peptide-his fusion proteins. **a** The coding sequence of each peptide was obtained by annealing two complementary oligonucleotides and cloned into a pGEX-His vector for fusion and expression with an N-terminal GST tag and a C-terminal 6× His tag. **b** The expression of GST-peptide-His fusion proteins was confirmed by Western blot with anti-GST and anti-6× His antibodies.

## Data Availability

The data that support the findings of this study are included within the article and its additional file.

## Abbreviations

GST: glutathione S-transferase
GSH: glutathione
SjSAPLP1: *Schistosoma japonicum* saposin-like protein 1
ELISA: enzyme-linked immunosorbent assay
ROC: receiver operating characteristic
AUC: area under curve
RLU: relative light unit
