# Supplementary figures and images for: Identification of linear epitopes in SjSP-13 of Schistosoma japonicum using a GST-peptide fusion protein microplate array
Source: Parasit Vectors. 2019 Oct 30;12:507. doi: 10.1186/s13071-019-3767-2 (PMC6822365; doi:10.1186/s13071-019-3767-2)

a

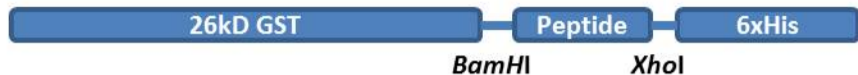

b

anti-GST Ab

p1 p2 p3 p4 p5 p6 p7 p8 p9 p10 p11 p12 p13 p14 p15 p16 p17

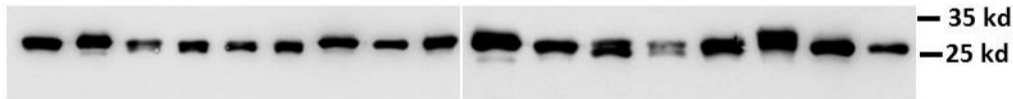anti- 6xHis Ab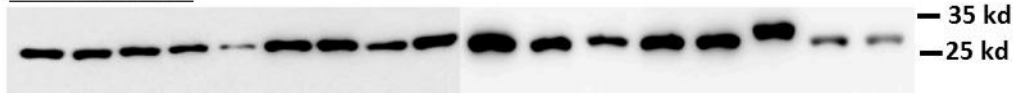

Supplement: Supplementary file 1 — Additional file 1: Figure S1. Construction of GST-peptide-his fusion proteins. a The coding sequence of each peptide was obtained by annealing two complementary oligonucleotides and cloned into a pGEX-His vector for fusion and expression with an N-terminal GST tag and a C-terminal 6× His tag. b The expression of GST-peptide-His fusion proteins was confirmed by Western blot with anti-GST and anti-6× His antibodies. [file 13071_2019_3767_MOESM1_ESM.pdf]
